# Limited ‘heft’ of weight-based outcomes in predicting influenza A virus disease severity in ferrets

**DOI:** 10.1371/journal.pcbi.1014210

**Published:** 2026-05-08

**Authors:** Troy J. Kieran, Taronna R. Maines, Jessica A. Belser

**Affiliations:** Immunology and Pathogenesis Branch, Influenza Division, National Center for Immunization and Respiratory Diseases, Centers for Disease Control and Prevention, Atlanta, Georgia; Georgia Institute of Technology, UNITED STATES OF AMERICA

## Abstract

Studies evaluating viral pathogenicity in small mammalian models often quantify disease severity using the magnitudes of temperature rise and weight loss post-challenge. However, no rigorous assessment on the transformation of serially collected data into features suitable for predictive models has been conducted. Using data aggregated from ferrets inoculated with a diverse panel of influenza A viruses (IAV) spanning a broad range of clinical outcomes, we assessed statistical correlations and predictive performance of temperature and weight loss, summarized by conventional and novel approaches. Conventional summary metrics (peak values or area under the curve) were weak and inconsistent correlates of overall disease severity and viral titers. Novel dynamic weight metrics capturing onset, duration, slope, and volatility over 14 days showed lower coefficients of variation than conventional summary approaches. However, inclusion of novel metrics did not meaningfully improve the predictive performance of machine learning models for disease severity outcomes in IAV-inoculated ferrets. Mixed-effects models indicated that weight loss post-IAV infection is driven by time and viral burden, with temperature contributing little additional information. Collectively, these findings support that derived metrics are at least comparable, if not enhanced, to conventional summaries for data science analyses of serially generated clinical data from *in vivo* pathogen studies. However, because pathogen disease severity in mammals is multifactorial, models that rely solely on weight and temperature metrics without additional quantitative measures of clinical perturbation within-host are unlikely to achieve strong predictive performance.

## Author summary

Viral pathogens are often studied in small mammals, where daily body temperature and body weight are routinely tracked to gauge disease severity. However, machine-learning efforts using these *in vivo* records have struggled to predict influenza morbidity reliably, raising a key question: is this due to inherent limitations in temperature and weight measurements, or in how these serial measurements are converted into model-ready features? We addressed this gap by analyzing two standard morbidity readouts (temperature rise and weight loss) using daily records from more than 800 ferrets infected with over 100 diverse influenza A virus strains (human, avian, and swine origin), forming one of the largest datasets of its kind. We systematically tested common summary metrics, identified which are most variable across infections, and introduced new derived metrics designed to better capture informative patterns in longitudinal trajectories. Combining statistical evaluation with machine-learning experiments, we show that careful feature construction from serially collected *in vivo* data can meaningfully affect predictive performance and interpretation. Because many pathogens and mammalian models generate similar serially-collected records, our methodological approach and results are broadly transferable, especially for researchers aiming to repurpose serial *in vivo* measurements for data-science and predictive modeling.

## Introduction

Most small mammalian models used to study viral pathogens often exhibit some degree of fever and/or weight loss following viral inoculation, depending on the virus [1], where the magnitude and duration of these clinical signs can range from mild and transient, to severe and persistent over the entirety of the observation period. Laboratory models that recapitulate these clinical signs can provide translatable insights relevant to human health. For example, influenza A virus (IAV) infection in humans is associated with a wide range of clinical perturbations, the severity and duration of which are influenced by a multitude of viral and host factors [2]. Experimentally inoculated ferrets can recapitulate many of these clinical signs, supporting the use of ferrets as a surrogate model to assess the pathogenicity of both seasonal circulating IAV strains as well as novel and emerging IAV from zoonotic reservoirs with pandemic potential [3]. IAV-associated disease severity (morbidity) in ferrets is typically captured by daily temperature and weight measurements which represent standard clinical parameters captured in IAV pathotyping studies conducted in ferrets. These data are considered in risk assessment rubrics (e.g., the CDC Influenza Risk Assessment Tool) to support pandemic preparedness [4,5]. Furthermore, comparative studies, such as assessing efficacy of a vaccine or antiviral treatment, perturbation of host innate and/or adaptive immune responses, or investigating differences between recombinant or reassortant IAV, consider modulation of clinical signs between experimental groups as a key outcome when determining relative differences in disease severity [6–8]. However, despite the common use of these parameters in the field, the relative predictive utility and statistical strength of morbidity measurements in the context of pandemic IAV risk assessment have not been rigorously assessed.

Perturbations to temperature and weight are typically analyzed and reported as normalized peak values relative to baseline over the observation period; area-under-the-curve (AUC) is an additional analytic approach that is often reported [1]. These clinical data, notably weight loss, are typically linked to disease severity, and are critical inclusions of humane euthanasia scoring for many acute and chronic disease models in numerous species [3,9]. Among IAV-inoculated ferrets, selected derived quantities from serially-collected *in vitro* replication data and *in vivo* viral titer data have demonstrated improved statistical correlations with key experimental outcomes, and heightened utility in machine learning (ML) predictive models, relative to conventional metrics [10–12]. However, to date, efforts to generate novel metrics have utilized viral titer measurements only, due to a paucity of aggregated datasets with sufficient diversity of pathogenic outcomes to study.

Prior studies utilizing data generated from IAV-inoculated ferrets have found that ML algorithms for binary outcomes of lethality or virus transmission can be highly predictive [12–14]. The high performance of these models is linked with extensive investigation into the most appropriate way in each setting to distill serially-collected data into discrete variables appropriate as features in ML settings [15]. However, prior ML models predicting disease severity (utilizing a binary percentage weight loss) have consistently underperformed [13,14]. These models have been limited to using peak values of clinical signs as features and outcome variables without extensive analyses of how the role derived quantities from these serially collected observations could improve the rigor of these data. It is thus unclear if the relatively poor predictive value of morbidity measurements to date reflects the quality of data itself, or the way in which these data are analyzed once collected.

To determine best practices for interpreting serially collected morbidity data from a pathogen-animal model, as an illustrative example, we examined IAV risk assessment studies in ferrets from aggregated daily weight loss and temperature readings from 832 ferrets inoculated with 113 unique IAV during previously conducted risk assessment studies over 25 years [16]. We analyzed which parameters show the greatest volatility across diverse IAV infections and then developed additional derived metrics to determine whether new features could offer comparable or improved predictive value in ML models. Our findings support that slope-based and volatility-focused summary measures of serially collected morbidity data provide meaningful complementary information and enhance traditional reporting approaches used in pandemic risk assessment, while highlighting that these clinical data alone are insufficient to robustly predict severe disease outcomes. Further, this methodological approach is highly applicable and translatable to other pathogens and mammalian species, and for data science applications utilizing serially-collected *in vivo* data.

## Results

### Assessment of utilizing peak recorded values to quantify morbidity

**Fig 1:**
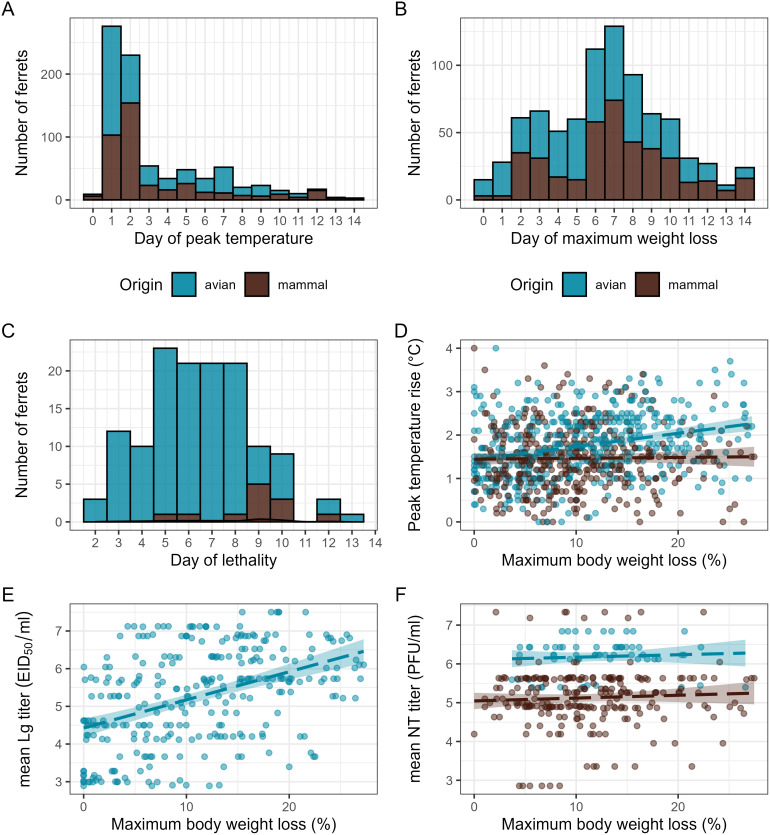
Timing of clinical signs and correlation of weight loss with viral titers in ferrets inoculated with diverse IAV. A, day of peak temperature rise over pre-inoculation baseline between days 0-14 p.i. among IAV-inoculated ferrets (n = 820). B, day of maximum weight loss under pre-inoculation baseline between days 0-14 p.i. among IAV-inoculated ferrets (n = 817). C, day of IAV-inoculated ferrets reaching humane endpoints between days 0-14 p.i. (n = 134). D, linear correlation between maximum weight loss and peak temperature rise (n = 829). Correlation lines are shown for avian-origin IAV (r = 0.29 [0.19, 0.39], p = 3.29e-8) or mammalian-origin IAV (r = -0.05 [-0.17, 0.07], p = 0.41). E, correlation (r = 0.43 [0.33, 0.52], p = 2.0e-14) between per-ferret maximum weight loss and mean per-virus log_10_ lung (Lg) titer among ferrets inoculated with avian-origin IAV using egg titration for infectious virus quantification (n = 291). F, correlations between per-ferret maximum body weight loss and mean per-virus log_10_ nasal turbinate (NT) titer among ferrets inoculated with IAV using cell titration for infectious virus quantification (n = 223) when stratified by IAV host origin. Correlation lines are shown for avian-origin IAV (r = 0.07 [-0.18, 0.31], p = 0.57) or mammalian-origin IAV (r = 0.06 [-0.07, 0.18], p = 0.39). For scatterplots, dots represent individual ferrets, and the dotted line represents Pearson linear correlation; gray shading represents 95% confidence interval. Color denotes host origin of each IAV (blue is avian origin, brown is mammalian origin). Additional supporting Pearson correlations are included in S1 Table.

Ferrets inoculated with IAV typically exhibit temperature rise and weight loss relative to pre-inoculation baselines over a 14-day p.i. observation period; measurable changes were detected in all but 1.8% (n = 15/832) and 1.1% (n = 9/829) ferrets for weight and temperature, respectively. Peak changes are frequently utilized to report perturbation magnitude during IAV infection (Table 1). In agreement with prior analyses [1], among IAV-inoculated ferrets with temperature rises from days 1–14 p.i., 61.7% (506/820) had peak values on days 1 or 2 p.i., and 78.3% (642/820) within the first 5 days p.i. (Fig 1A). These peaks were generally similar for avian-origin or mammalian-origin viruses. In contrast, peak weight loss during the 14-day observation period showed substantial variability; while highest frequency was on day 7 p.i. for both avian and mammalian-origin viruses, this only represented 15.8% of ferrets (129/817), with at least 50 reaching peak weight loss on each day between day 2–10 p.i. (Fig 1B). Similarly, lethal outcomes (frequently associated with neurological complications or weight-based humane endpoint criteria) were reported between days 2–13 p.i. (Fig 1C), supporting that severe disease can be detected throughout the acute phase of infection with diverse IAV. These records indicate that inoculation of ferrets with both avian and mammalian-origin IAV can lead to similar timing of peak morbidity parameters, though temperature peaks are more constrained relative to the higher variability of weight loss.

**Table 1:** Conventional and novel variables examined in this study.

Category	Name in study	Description	Biological meaning in IAV-infected ferrets
Peak value	max_wt_loss_pct	Percent maximum weight loss between days 1–14 p.i.	Ferrets exhibiting severe disease typically lose more weight than ferrets exhibiting mild to moderate disease; maximum weight loss ≥25% represents a humane endpoint.
	tp_max_5	°C rise in body temperature between days 1–5 p.i.	Ferrets often run fevers early post-infection regardless of overall disease severity
Area under the curve (AUC)	AUC_1_wt:AUC_x_wt	AUC of normalized weight loss days 1-x p.i.	Ferrets that fall below baseline weight for a longer period of time will have a higher AUC than ferrets where weight loss is transient before recovery
	AUC_1_tp:AUC_x_tp	AUC of normalized temperature days 1-x p.i.	Ferrets that have sustained fevers for multiple days will have a higher AUC than ferrets where fever is transient
Onset and duration	wt_onset_xd_y	First day when weight change from baseline exceeds a threshold y for x days (d)	Ferrets that lose more weight often start losing that weight faster than ferrets exhibiting mild to moderate disease
	wt_days_x_dy	Total number of days x percentage of weight loss is met and/or exceeded days 1-y p.i.	Weight loss may persist for a longer duration in ferrets exhibiting more severe disease relative to ferrets with mild infection
Slope	wt_loss_slope_max	Slope of peak weight loss relative to baseline	Ferrets that lose weight more rapidly p.i. will have a lower slope than ferrets where peak weight loss is recorded later during infection
Volatility	RMSSD	Root mean square of successive differences	Ferrets that have higher day to day variability in weight loss/temperature readings will have a higher RMSSD than ferrets where day to day variability is reduced
	ACF1	Lag-1 autocorrelation	Ferrets that have weight/temperature values one day that are dissimilar to the day before will have a lower ACF1 than ferrets with a smoother day to day trajectory

Temperature and weight loss represent multifactorial traits that may be influenced by various viral and host processes; peak weight loss exhibited low correlations with peak temperature rise across the entire dataset (r = 0.18 [0.12, 0.25], p = 1.2e-7) or when viruses were separated by host origin (Fig 1D). Correlations between peak temperature rise and viral titer measurements during the acute phase of infection were inconsistent [14]. Similarly, weight loss correlations with viral titers varied; maximum weight loss had a moderate correlation with mean lower respiratory tract (lung) viral titers day 3 p.i. among avian-origin IAV titered in eggs (Fig 1E), but correlations were overall reduced or absent with upper respiratory tract viral replication regardless of the metric evaluated (day 3 nasal turbinate (NT) titers, mean nasal wash (NW) titers, day 1 NW titers) (Fig 1F and S1 Table). Taken together, these analyses support that peak values of temperature rise or weight loss offer only modest to low correlations with each other or infectious viral loads, limiting their predictive value regarding disease severity in IAV-inoculated ferrets.

### Assessment of utilizing disease onset and duration-based values to quantify morbidity

Peak values do not capture the overall duration of clinical signs in IAV-inoculated ferrets. Area-under-the-curve (AUC) values are commonly used to assess the duration and magnitude of morbidity during a 14-day period (Table 1). However, no systematic evaluation has been performed assessing the time span that best encapsulates morbidity during acute infection. As such, we calculated per-ferret AUC for normalized temperature rise and weight loss, starting at baseline and terminating daily from 1-14 p.i. With few exceptions, these sequential AUC parameters did not correlate with virus replication metrics in the upper or lower respiratory tract relative to the peak values assessed above (S2-S3 Tables). This suggests that conventional AUC parameters capturing weight loss and temperature rise do not provide consistent, meaningful improvements over peak recorded values.

**Fig 2:**
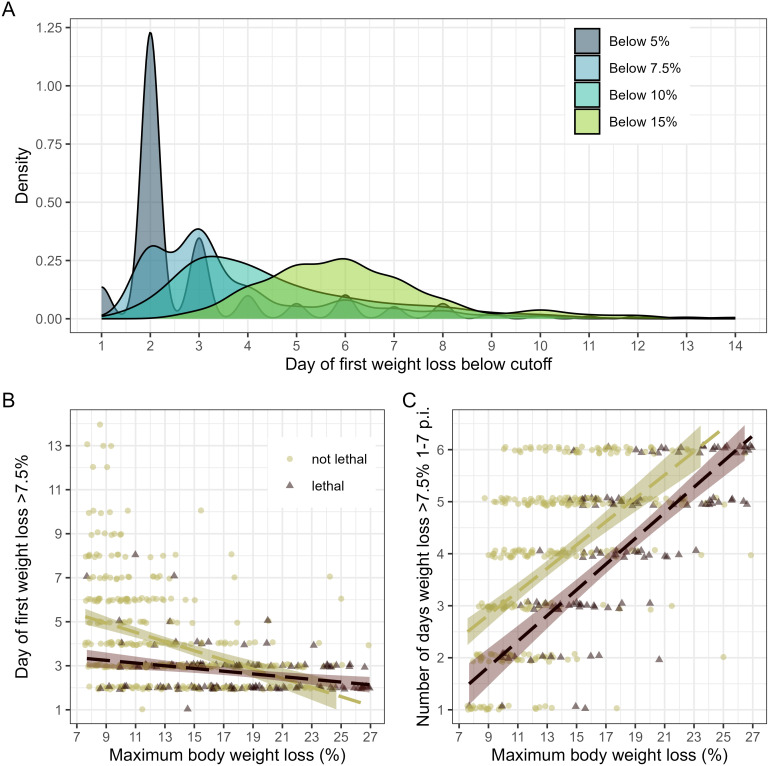
Timing and duration of weight loss in IAV inoculated ferrets. A, Density plot depicting the day range of first recorded normalized weight loss at or below the cutoff percentage specified between days 1-14 p.i. in IAV-inoculated ferrets (n = 832). B, correlation between maximum weight loss and day of first weight loss recorded in excess of 7.5% among IAV-inoculated ferrets. Correlation lines are shown for ferrets with lethal outcomes (r = -0.27 [-0.42, -0.11], p = 1.49e-3) or survival outcomes (r = -0.38 [-0.46, -0.29], p = 5.27e-14) during the 14-day observation period. C, linear correlation between maximum weight loss and number of days weight loss was recorded in excess of 7.5% among IAV-inoculated ferrets. Correlation lines are shown for ferrets with lethal outcomes (r = 0.78 [0.71, 0.84], p < 2.2e-16) or survival outcomes (r = 0.82 [0.79, 0.84], p < 2.2e-16) during the 14-day observation period. For scatterplots, dots represent individual ferrets, and the dotted line represents Pearson linear correlation; shading represents 95% confidence interval. Color denotes lethal (maroon) or survival (olive green) outcome of each ferret. Additional supporting Pearson correlations are included in S4-S5 Tables.

As weight loss exhibited high temporal variability (Fig 1B), we examined the first day each ferret exceeded set weight loss thresholds from the pre-inoculation baseline. As expected, greater weight loss takes more days to occur, as median observation days for ferrets with higher overall weight loss (either ≥5%, 7.5%, 10%, or 15% total weight loss) were 2, 3, 4, and 6 p.i. for each threshold, respectively (Fig 2A). Furthermore, ferrets with greater weight loss met these thresholds over a wider time range than ferrets exhibiting less overall weight loss (such that the median observation day represented over 57% of animals with weight loss ≥5% but represented less than 30% of ferrets with weight loss ≥10% or ≥15%). These observations support that greater maximum weight loss can be associated with a dynamic range of timing (Fig 1B) and onset (Fig 2A) not observed during mild disease.

Next, we examined how key morbidity thresholds were reached relative to overall peak values during the 14-day observation period. Across the dataset, statistically significant correlations (r > 0.4, p < 2.2e-16) between maximum weight loss and the day ferrets first reached weight loss thresholds ≥5%, ≥ 7.5%, or ≥10%) were observed, supporting that ferrets reaching higher maximum weight loss p.i. started losing that weight more quickly (Fig 2B and S4 Table). When stratified by lethal outcome, correlations were stronger among surviving ferrets, possibly due to a wider range of days when weight loss thresholds were met compared to ferrets with lethal outcomes (Fig 2B). Comparable results emerged when analyzing the first day ferrets reached specified weight loss thresholds over two consecutive days (S4 Table). Additionally, we determined the number of days ferrets recorded weight loss below a specified threshold to assess disease severity persistence during the acute phase of infection. Independent of lethal outcome, ferrets losing ≥7.5% of pre-inoculation weight for more days (days 1–7 p.i.) were associated with higher peak weight loss over the entire 14-day period (Fig 2C and S5 Table). Furthermore, weight loss among ferrets reaching lethal outcomes was persistently low for more days (mean and median of 4.01 and 4 days, respectively) than those surviving the observation period (1.85 and 0 days, respectively). Comparable statistically significant results were found when defining persistent weight loss as ≥5% or ≥10%, or when considering the acute phase as the first 5 days p.i. (S5-S6 Tables). Collectively, these findings support that variables capturing the onset and duration of morbidity (measured by weight loss) can be strongly associated with peak morbidity readings and disease outcomes.

### Generation of novel slope and volatility parameters to quantify morbidity

We assessed whether variable kinetic considerations during the 14-day observation period could yield discrete values with high statistical significance against clinical and/or virological measures of disease severity in IAV-inoculated ferrets. This involved selecting two fixed values and determining the slope of growth or decay or calculating a volatility value over the entirety of the observation period.

**Fig 3:**
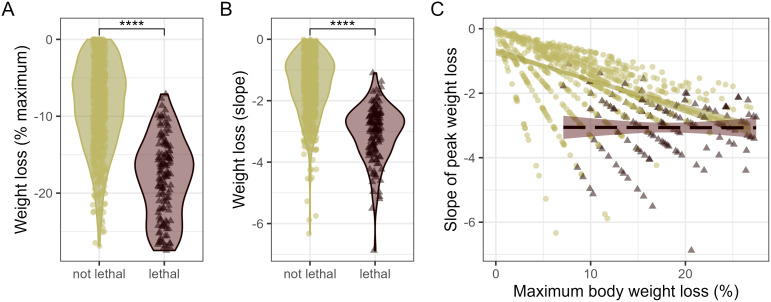
Relationship between maximum weight loss and weight loss slope in ferrets inoculated with IAV. A, percent maximum weight loss among ferrets exhibiting survival or lethal outcomes during the 14-day observation period. B, slope between baseline and percent maximum weight loss among ferrets exhibiting survival or lethal outcomes during the 14-day observation period. ****, relative statistical significance between groups shown is p < 1e-40 by Wilcoxon test (see S7 Table). C, Pearson linear correlation between maximum weight loss and slope of peak weight loss (n = 816). Correlation lines are shown for ferrets that exhibited lethal outcomes post-IAV inoculation (r = -0.004 [-0.17, 0.17], p = 0.9644) or ferrets that survived the IAV infection (r = -0.051 [-0.57, 0.46], p < 2.2e-16). Dots represent individual ferrets and are colored olive green or maroon to denote survival or lethal outcomes, respectively.

Leveraging the variable range of when peak weight loss values were detected, we calculated the slope of peak weight loss relative to baseline for each ferret (wt_loss_slope_max, Table 1). Among ferrets with lethal outcomes, maximum weight loss was significantly higher, and the slope of maximum weight loss was significantly lower, than ferrets that survived (Fig 3A- 3B). Unsurprisingly, peak weight loss was well-correlated with the slope of maximum weight loss (r = -0.59 [-0.64, 0.55], p < 2.2e-16), indicating that ferrets losing more weight had more negative slope values. However, when stratified by lethal outcome, this correlation existed only among survivors (Fig 3C), likely due to a wider dynamic range of peak weight loss in survivors compared to those with lethal outcomes.

**Fig 4:**
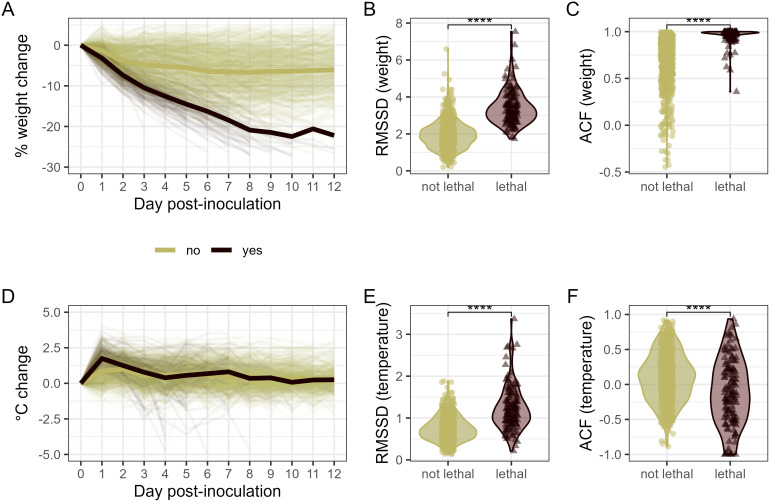
Assessment and quantification of volatility in clinical signs among ferrets inoculated with IAV. Daily percent weight change (A) or daily °C temperature change (D) from normalized baseline days 1-12 p.i. in ferrets inoculated with IAV that exhibited survival (tan lines) or lethal (red lines) outcomes. Thin lines represent individual ferrets; the thick line represents daily mean value per group. RMSSD (B, E) or ACF (C, F) calculated from daily weight change (B, C) or daily temperature readings (E, F) among ferrets exhibiting survival or lethal outcomes during the 14-day observation period. ****, relative statistical significance between groups shown is p < 1e-50 (B, C) or p < 1e-6 (E, F) by Wilcoxon test (see S7 Table). Dots represent individual ferrets and are colored olive green or maroon to denote survival or lethal outcomes, respectively.

Beyond quantifying differences in absolute measures of deviation from pre-inoculation baselines, we examined day-to-day variability in temperature and weight loss metrics to capture volatility in serially collected measurements. Ferret observations can show substantial within 24-hour variability (>15% weight loss or >3°C rise). While normalized temperature changes are more compressed than weight loss (in both day to day changes and patterns of overall change in animals that do or do not reach humane endpoints, Fig 4A and 4D), we hypothesized that ferrets with severe disease (higher peak morbidity and/or lethal outcomes) would exhibit greater volatility across daily measurements relative to ferrets with mild disease, measurable by a discrete value. Discrete volatility measures were calculated in two ways. First, by using the root mean square successive difference (RMSSD) to capture the magnitude of fluctuations in daily temperature and weight trajectories (mean absolute successive difference (MASD) was like RMSSD and data is not presented). Second, by assessing lag-1 autocorrelation (ACF1), to measure the linear relationship between an observation and its immediate predecessor (i.e., how similar the value today is to the previous day) (Table 1).

The difference in RMSSD based on daily weight (Fig 4B) or temperature (Fig 4E) between days 0–14 p.i. was highly significant when ferrets were stratified by lethal outcome, with ferrets exhibiting severe disease having higher RMSSD values, indicating lower overall volatility of these variables in IAV infected ferrets. RMSSD from weight measurements also exhibited a strong linear correlation with peak weight loss (r = 0.60 [0.56, 0.65], p < 2.2e-16), as did RMSSD from temperature measurements with peak temperature (r = 0.50 [0.44, 0.55], p < 2.2e-16). Similarly, ACF1 based on daily weight (Fig 4C) or temperature (Fig 4F) between days 0–14 p.i. was highly significant when ferrets were stratified by lethal outcome. For this metric, higher autocorrelation equals a smoother trajectory over time, whereas lower values indicate greater day‑to‑day variability. Like RMSSD, ACF1 from weight measurements exhibited a strong linear correlation with peak weight loss (r = 0.58 [0.53, 0.62], p < 2.2e-16); interestingly, linear correlations between ACF1 from peak temperature were not robust (r = -0.10 [-0.17, -0.03], p = 0.0032), supporting that ACF1 from temperature data was not as effective a parameter. Taken together, these results support that IAV infected ferrets that exhibited lethal outcomes had overall less volatility in clinical measurements compared with ferrets that survived the 14-day infection.

### Clinical parameter-based variables poorly predict severe morbidity outcomes

We previously demonstrated that ML algorithms trained on data from ferrets inoculated with diverse IAV strains could predict lethal outcomes, but not morbidity (defined as weight loss >14.5% of pre-inoculation weight) [13,14]. To explore if novel parameters offered greater predictive value than conventional peak value metrics, we assessed whether slope or volatility metrics from temperature and weight loss data could enhance an underperforming disease severity/morbidity predictive ML model. First, we examined these parameters in elastic net models to predict the high weight loss category (wt_loss_high) from the original base model (that included nasal wash titer AUC days 1–6 per ferret (AUC_6_f), peak temperature days 1–5 (temp_5), HPAI_MBAA, RBS, PA, and virus HA-NA subtype as features, see Methods for full description) [13], then trained the ML model and evaluated performance via Matthew’s Correlation Coefficient (MCC). Replacing peak temperature days 1–5 with either temperature RMSSD or ACF1 retained temperature as a highly ranked feature, but each model underperformed relative to the base model (S8 Table). Every slope or volatility metric based on weight loss data modifying the base model became the highest ranked variable (unsurprising given the outcome is based on peak weight loss). Despite this, limited to no model improvement over the base model was observed via MCC with all parameters (S9 Table); a model with weight ACF1 had higher performance metrics than the base model (0.63 vs 0.43 MCC), whereas slope showed a marginal improvement and weight RMSSD underperformed.

Previous underperforming ML models predicting disease severity utilized a weight loss cutoff (>14.5%) as the outcome variable [13,14]. We next assessed if slope or volatility metrics would improve model performance if treated as outcome variables rather than features. Thus, we retained all base model features and compared performance where the outcome variable was weight loss-based slope, RMSSD, or ACF1 against the high weight loss category. Outcome variables were classified into a binary yes (high third of data) and no (remaining two thirds). However, modulating the outcome variable did not substantially change feature rankings and importance (S8 Table), and all models with new outcome variables performed poorly relative to the base model using a conventional weight loss outcome (S9 Table). Collectively, these results support that ML models utilizing sequence and virus titer-based features to predict weight loss-based outcomes underperformed relative to lethality-based models, even when transforming weight loss data to capture dynamic parameters rather than peak values during the observation period.

### Clinical parameter-based metrics can exhibit high coefficients of variation

**Fig 5:**
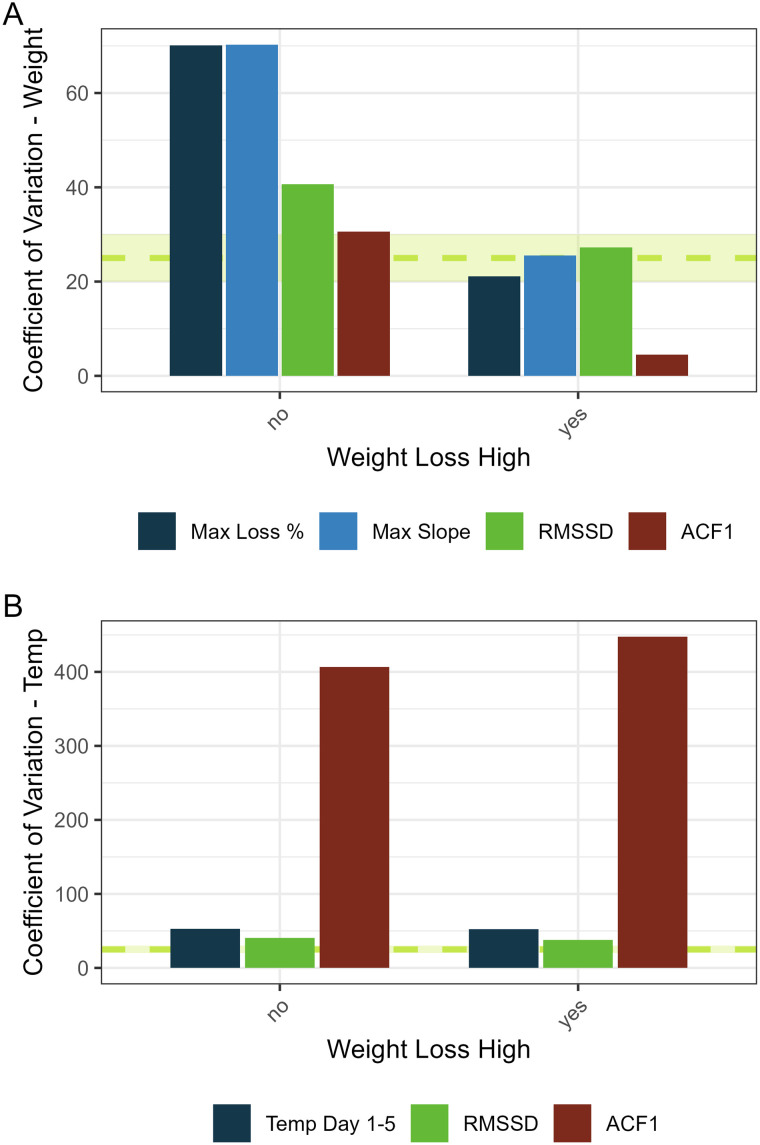
Coefficient of variation of variables for binary weight loss categories. A, Coefficient of variation (as a percentage) for maximum percent weight loss (conventional parameter) or maximum slope, RMSSD, or ACF1 calculated from daily normalized baseline weight between days 1-14 p.i. for each binary outcome category of weight loss high (high third of data). B, Coefficient of variation (as a percentage) for maximum temperature rise days 1-5 p.i. (conventional parameter) or RMSSD or ACF1 calculated from daily normalized change in temperature rise days 1-14 p.i. for each binary outcome category of weight loss high. Dashed line with shading represents 25% + /- 5% as a generic reference of biological variation.

Parameters that exhibit a low coefficient of variation are considered more stable and less variable relative to the mean [17], which can improve predictive models for parameters that exhibit increased variation. To better understand why features based on clinical data do not offer high value in predictive models, we assessed the coefficient of variation of weight loss or temperature for each discrete parameter on a per-ferret basis. Among parameters based on serially collected weight loss data, maximum slope exhibited comparable variance relative to the conventional peak weight loss parameter (Fig 5A). Interestingly, both RMSSD and ACF1 parameters exhibited generally similar reduced variance, with ACF1 slightly more consistent than RMSSD. However, when considering parameters based on serially collected temperature data, volatility measures differed greatly, with RMSSD like a conventional peak temperature parameter, but ACF1 exhibiting variation an order of magnitude higher (Fig 5B). These results suggest that slope and volatility metrics based on weight loss, despite not being capable of improving current predictive models, may nonetheless represent features that are more stable, less variable, and more consistent across observations relative to conventional metrics.

### Linear mixed-effects models to examine variable relationships and influence

To more rigorously assess relationships between clinical parameters, we constructed linear mixed effects models that considered weight, temperature, viral titer, and temporal values (day), while accounting for virus and per-ferret within-virus variation, to examine these metrics in relation to each other. Results are presented in Table 2. We first established linear mixed‑effects models that considered relationships between weight loss, temperature, and viral titer, utilizing conventional parameters as outcome variables (daily 1–14 weight loss, days 1–14 temperature change, days 1–9 NW viral titer) on a per-day basis. Overall, these models suggest coordinated trajectories in which viral burden and time are key drivers of weight loss, with temperature showing only modest, largely time‑related changes. These models revealed that weight decreased markedly over time (about 0.97 per day, p < 2e‑16) and was lower when viral titers were higher, while slightly higher temperatures were associated with higher weight. Random effects were present between‑virus variability (intercept standard deviation (SD) = 1.36, day‑slope SD = 0.88) with larger heterogeneity across ferrets within viruses (intercept SD = 1.94, day‑slope SD = 0.67). Temperature decreased slightly over time, was modestly higher in heavier animals (beta = 0.010, p = 0.00059), and was not associated with titer and virus‑level variation was moderate (intercept SD = 0.56, slope SD = 0.10), with smaller ferret‑level slope variation (intercept SD = 0.45, slope SD = 0.035). Restricting to days 1–9 p.i., viral titer declined sharply with time (beta = −0.592 per day, p < 2e‑16), was lower in heavier animals (beta = −0.023, p = 8.8e‑15), and was not related to temperature (beta = 0.010, p = 0.50). Virus‑level random effects were substantial (intercept SD = 1.28, slope SD = 0.19; intercept–slope correlation = −0.85), while ferret‑level variation was smaller (intercept SD = 0.41, slope SD = 0.034). Random‑effects indicated meaningful heterogeneity at the virus level (notably for viral titer, where viruses with higher baselines tended to decline faster) and individual differences within viruses, particularly in baseline weight and temperature; day‑to‑day slope differences were small at the ferret level for temperature and titer.

**Table 2:** Linear mixed effects models using conventional and novel morbidity metrics.

Outcome^*a*^	Body weight		Temperature		NW viral titer		Biological interpretation^*c*^
	beta^*b*^	p	beta	p	beta	p	
day	-0.97	<2e-16	-0.089	1.6e-12	-0.59	<2e-16	Body weight and viral titer decline more over time than modest temperature decline
weight (daily %)			0.01	5.9e-4	-0.023	8.8e-15	Higher weight loss is associated with slightly higher temperature and lower titer
temperature (daily rise)	0.11	5.4e-3			0.01	0.5	Temperature is modestly higher with more weight loss
titer (daily titer)	-0.2	6.5e-14	-0.003	0.77			Higher weight loss (but not temperature) is associated with lower viral titers
weight (RMSSD)			0.09	<2e-11	-0.003	0.51	Higher temperature volatility detected in ferrets with greater weight volatility
temp (RMSSD)	0.62	<1e-11			-0.24	0.98	Temperature volatility increases slightly when weight volatility is higher
titer (AUC_6)	0.006	>0.42	-0.003	>0.42			Weight loss and fever do not predict viral titer

^*a*^Outcome was defined using conventional metrics on a per-day, per-ferret basis (first four rows) or novel metrics on a per-ferret basis (last three rows). Titer measurements were determined using infectious virus recovered from ferret NW specimens collected days 1–9 p.i. (daily titer) or 1–6 p.i. (AUC_6). ^*b*^beta is the test statistic that estimates the amount of change being observed. ^*c*^Describes general trends in temporal relationships between different features and outcome variables.

We further investigated if weight-based RMSSD could provide additional insight as a surrogate outcome variable by substituting weight and temperature for their respective RMSSD values, and exchanging titer for the AUC of nasal wash titer between days 1–6 p.i. Across mixed‑effects models with virus as a random intercept, weight variability was strongly and positively associated with temperature variability (beta 0.62, p < 1e-11), and the reciprocal model showed a smaller but still significant positive association of temperature variability with weight variability (beta 0.09, p < 2e-11). In both models, titer AUC was not associated with either outcome (p > 0.42). When modeling titer AUC directly, neither weight nor temperature variability predicted titer burden (p = 0.51 and p = 0.98, respectively), while viruses differed substantially in titer AUC (random intercept SD 3.46). Virus‑level heterogeneity in variability outcomes was modest (SD 0.45 for weight variability, 0.19 for temperature variability). Overall, variability in weight and temperature covaried, whereas titer burden showed no detectable relationship with either variability metric and exhibited pronounced differences across viruses. Taken together, these findings support the strong interplay of metrics capturing morbidity and further support the utility of measures of volatility as a valuable additional feature to understand disease burden and severity.

## Discussion

*In vivo* experimentation permits the study of multifactorial traits (including but not limited to disease severity following pathogen challenge) that cannot be modeled outside of a living host. While it is unlikely that *in silico* models will fully replace the use of animals in these settings, predictive models leveraging results from previously conducted studies can identify trends in data that may not be apparent outside of the aggregate, offer improvements to summary metrics derived from these data, and contribute to efforts reducing overall animal usage by creating predictive models when feature and outcome variables suitable for this work are identified. However, working with *in vivo* data represents a particular challenge when conducting this work, due to the serial nature of many virological and clinical parameters collected that necessitate manipulation prior to use in statistical and modelling algorithms, and the relatively low sample sizes available for training and testing predictive models [15,18]. Nonetheless, successful predictive models have been established utilizing a range of virological and clinical data following *in vivo* experimentation with multiple viral pathogens (such as IAV, SARS-CoV-2, ebola virus, and rabies virus) in different animal models (including mice, ferrets, and non-human primates) [13,19–22], underscoring the utility of these datasets despite the challenges presented. Reporting relative disease severity during experimental IAV infection in small mammalian models represents a routine analysis in activities assessing viral pathogenesis, but no dedicated effort has been conducted to determine the most rigorous and predictive discrete measures that may be computed from this serial data. Given the critical role disease severity considerations contribute to IAV risk assessment rubrics [4,5], understanding which summary metrics most robustly capture disease severity in virus-infected animals, and assessing if standard experimental protocols collect a sufficient diversity of data to interpret and predict this highly multifactorial outcome, represents a necessary effort that can be translatable to other animal and disease model systems.

This study focused on weight loss and temperature data, as these are the two most common parameters that are collected and reported in the most uniform way across institutions performing IAV pathogenesis studies worldwide [23], in addition to a diversity of other pathogen studies conducted in mammalian models [1]. Other clinical observations (such as animal activity level) may also be assessed in a serial fashion during experimentation and examined in the aggregate [24], but lethargy was not considered here due to the potential for subjective confounding by investigator and a diversity of scoring approaches utilized across different institutions. Depending on the experimental design and informed by the multifactorial nature of viral pathogenesis, studies may include a multitude of additional data metrics that may be included in machine learning, logistic regression, or other models (including noting frequency and duration of rhinorrhea, diarrhea, and sneezing, observation of inappetence and hydration, quantification of host responses in discrete tissues, modulation of lymphohematopoietic parameters, histopathology scores, among others) [19,24–26]. As such, while weight loss and temperature rise are considered as meaningful surrogates capturing disease severity, given the panoply of host processes that may be perturbed during acute IAV infection, it is not surprising that these two variables offer an incomplete set of features for predictive model use.

Across models and analyses (both using IAV ferret data specifically and pathogen mammalian disease models in general), clinical parameter-based features have in many instances struggled to predict morbidity reliably [13,14,27]. Elastic net and machine learning models trained to classify high weight loss (≥14.5%) performed similarly or worse when conventional peak metrics were replaced by slope or volatility measures for temperature or weight, even though these novel features often ranked highly. The one notable exception was weight based ACF1, which improved Matthew’s Correlation Coefficient to 0.63 compared to the base model of 0.43. In contrast, weight RMSSD underperformed and slope yielded only marginal gains. Reframing the outcome by predicting slope or volatility (instead of features) did not materially improve feature importance or model performance relative to the traditional weight loss outcome. These results suggest that for morbidity endpoints that include weight loss thresholds, the available clinical features (whether peak, slope, or volatility) may not provide meaningful predictive value, and model gains are limited by the outcome definition and the inherent variability in these measures.

Complimentary analyses show why prediction of morbidity outcomes may be difficult. Coefficient of variation assessments indicated that weight loss volatility metrics (RMSSD and ACF1) were generally more stable than peak weight loss, whereas temperature volatility showed heterogeneous behavior, with ACF1 markedly more variable than RMSSD and peak temperature. Mixed-effects models support expected biological relationships with weight loss increasing over time, and higher weight loss associated with higher viral titers in nasal wash specimens, and (to a lesser extent) increased temperature. Additionally, temperature declined over time and was not meaningfully associated with viral titer, while titer dropped sharply during the early infection days with substantial virus-level variability. These models further showed that weight and temperature variability covary, while titer burden from nasal wash specimens (AUC) was unrelated to either metric but it differed between viruses. Taken together, these findings indicate that volatility captures meaningful aspects of morbidity dynamics, but on its own does not overcome the limited predictive value of clinical parameters for this outcome. Future work should consider alternative outcome definitions, optimized thresholds, and additional biological features to better quantify and predict severe disease. This could include the use of viral titer data in linear mixed-effects models other than serially-collected nasal wash specimens, as infectious viral load in tissues collected during the acute phase of infection have shown predictive value in ML settings [14,19].

Machine learning offers a valuable complementary tool to conventional statistical and analytical workflows to assess the relative predictive strength of different metrics derived from *in vivo* experimentation. Feature selection and feature importance assessments can identify which parameters offer highest predictive value for a particular outcome. Using this approach, we have identified specific features that offer high predictive value for both lethal outcomes in ferrets and transmission outcomes between ferrets following IAV inoculation [12–14]; other multivariate analyses have also investigated virulence in mammalian models, as defined by lethal outcome [28]. However, models assessing weight loss-based outcomes have consistently underperformed when features included virological sequence data with or without clinical data or viral titer data. Our finding that slope or volatility metrics based on weight loss data did not improve predictive ML models when these variables were used, as either features or outcome variables, strongly supports that conventional metrics captured during current risk assessment work (inclusive of molecular, virological, and clinical parameters) are insufficient to provide parameters that can predict disease severity. That said, we investigated several variables but did not evaluate the thresholds used to define binary categories. We previously showed that predictive value can depend on the chosen cutoff [12], so both the conventional and novel parameters studied here may offer stronger prediction with further optimization of meaningful thresholds.

This study had several limitations. Source data was limited to experimental records from one research group. Because serially collected temperature and weight data is not commonly reported in the field, no external validation of ML models could be conducted. While it is likely that the novel metrics presented here could be applied to data generated in other mammalian models (such as mice), comparable weight loss data from a second species was not assessed. Novel metrics examined focused primarily on the onset, duration, and magnitude of establishment of severe disease, but did not examine recovery-based metrics due to limited daily sampling of clinical signs among some animals with mild disease throughout the entire 14-day observation period which limited robust computation of these parameters. However, it is possible that the approaches described here could nonetheless be applied for recovery-based metrics. Normalized data were utilized to generate all derived quantities and perform all analyses, due to high baseline variability in both body weight (732.2-2055.8g) and temperature (35.9-40.1°C) at the time of inoculation in ferrets; for this reason quantification of absolute change in g or °C was not considered. Because humane endpoints consider both weight-bound and non-weight-bound measurements, some IAV-infected ferrets were humanely euthanized during the experimental period with weight values that in isolation would not necessarily indicate severe disease (Fig 1C), influencing many of the weight loss-based parameters investigated. That said, the high diversity of IAV strains represented in this study [29], and the consistent protocol governing generation and collection of all data examined during the >25-year span these experiments were conducted, supports a robust dataset suitable for rigorous investigation of key clinical signs captured and analyzed during standard viral pathogenesis studies.

Researchers have an ethical obligation to ensure studies relying on *in vivo* experimentation are performed and interpreted to the highest standard; one approach in support of this effort is to critically examine historical data to identify if existing practices can be improved upon to further scientific goals. Using aggregated IAV challenge data from the ferret model as a representative example, we found that conventional metrics used throughout viral pathogen studies conducted in mammalian models can be subject to undesirable features. For example, temperature peaks may be compressed, and peak weight-loss values vary widely, correlating poorly with the key outcomes the clinical data are purported to support. Generating novel metrics that consider not just peak values but the timing, growth, and volatility of these serially collected measurements represents a needed effort in the field to ensure that studies performed *in vivo* are meaningfully and accurately reported in the literature. While the clinical signs used in this study were insufficient to predict or distinguish outcomes of severe disease as defined using a weight loss outcome variable, this work nonetheless highlights the need to capture and contextualize additional diverse parameters as possible when assessing disease severity in viral pathogen studies (both virological and clinical, especially in the context of pathogen risk assessment), and consider novel metrics such as the ones presented here to capture the full dynamic nature of clinically-based observations.

## Methods

### Ethics statement

All analyses were conducted on previously performed animal work that was approved by the CDC Institutional Animal Care and Use Committee (IACUC) in an AAALAC International-accredited facility; data were aggregated for this analysis from these published studies [16].

### Viruses and ferret inoculation

Influenza A viruses (133 strains, inclusive of H1, H2, H3, H5, H7, and H9 subtypes) were propagated in 10–11 day old embryonated hen’s eggs or Madin Darby Canine Kidney (MDCK) cells as previously described [30]. All manipulations with live virus were performed at either biosafety level 2 or biosafety level 3 containment, including enhancements, as required by the U.S. Department of Agriculture and the Federal Select Agent Program depending on the requirements of each viral strain [31]. Host origin of each virus was indicated as avian (isolated from an avian host or originating from an avian species during a zoonotic spillover to humans) or mammalian (inclusive of human-origin, swine-origin, canine-origin, or variant [human infection with swine-origin virus]). Predicted receptor binding preference (RBS, avian, human, or dual) and predicted polymerase activity (PA, avian or human) were defined based on hemagglutinin (HA) and polymerase basic 2 (PB2) amino acid residue identity as previously described [32]. Viruses with a multibasic amino acid (HPAI_MBAA) HA cleavage site were identified as yes or no.

No new animal work was conducted for this study. Male Fitch ferrets (5–12 months of age, from Triple F Farms [Sayre, PA] unless otherwise specified) were serologically negative to influenza A and B viruses circulating at the time of use. Prior to inoculation, a subcutaneous temperature transponder (IPTT-300, BMDS, Seaford, DE) was inserted into the dorsal space between the scapulae of each ferret to monitor body temperature. Ferrets (minimum n = 3/virus) were housed in HEPA-filtered Duo-Flo BioClean mobile environmental enclosures (Lab Products Inc, Seaford, DE) for all experiments. Ferrets were inoculated under anesthesia (25 mg/kg ketamine, 2 mg/kg xylazine, with or without 0.05 mg/kg atropine in the hamstring) by the intranasal route with a high dose (10^5^-10^7^ infectious units) of virus in a 1mL volume.

### Ferret experimentation and sample collection

No new animal work was conducted for this study. All ferret data consists of previously published records that have been aggregated for the analyses herein. Details describing experimental protocols used in aggregated data are described previously [16]. Briefly, previous animal work was conducted under standardized protocols as follows. Ferrets (n = 832 and n = 829 for weight and temperature measurements in the dataset, respectively) were observed between days 1–14 p.i. Every 24–48 hours, weight and temperature readings were recorded. Every 48 hours, nasal washes were collected under anesthesia as previously described [16], stored at -80°C, and subsequently titered in either embryonated hen’s eggs or MDCK cells to determine a 50% Egg Infectious Dose (EID_50_) titer or Plaque Forming Units (PFU) titer, respectively. Any ferret that lost >25% of pre-inoculation body weight, exhibited neurological complications, or pronounced lethargy [33] was humanely euthanized.

### Calculation of morbidity parameters

All analyses were conducted in R v4.4.0 using packages tidyverse v2.0.0 [34] and janitor v2.2.0 [35]. Daily weight loss and temperature measurements collected between days 0–14 p.i. were aggregated. Mean values from missing observations were interpolated with means when there was a recorded value flanking either side. Data were normalized using the baseline (day 0) temperature or weight reading as previously described [1]. Temperature and weight loss were normalized for each day on a per-ferret basis by subtracting the raw values from the baseline value. Additionally, normalized weight loss was multiplied by 100 to convert to a percent change. Sequential AUC was calculated with the trapezoid method per ferret for complete data observations without lethal events using DescTools v0.99.57 [36]. Maximum weight loss slope was calculated as the maximum weight loss percentage divided by the maximum weight loss day for each ferret. We identified the onset of the first day where the measurement met the threshold in the specified direction for at least k consecutive non-missing observations, determined via run-length encoding. We calculated volatility as the rate-normalized root mean square of successive differences (RMSSD) using the square root of the average of squared successive changes in the measurement divided by their time gaps, using only pairs with positive time gaps and non-missing values. We calculated robust volatility as the rate-normalized mean absolute successive difference (MASD) using the average absolute successive change in the measurement divided by its time gap, using only pairs with positive time gaps and non-missing values. We estimated lag-1 autocorrelation (ACF1) as the Pearson correlation between adjacent non-missing observations in the time series.

### Statistical and correlation analyses

All analyses were conducted in R v4.4.0. Base R stats was used to calculate Pearson product-moment correlations with or without adjustment for multiple comparisons by Holm’s method, and Wilcoxon Ranked Sum tests. Normality was assessed via Shapiro-Wilk and homogeneity of variance was assessed by Levene’s test using the car v3.1-3 package [37]. We calculate the coefficient of variation by first calculating the median and median absolute deviation (MAD) for each high weight loss (wt_loss_high) group and variable, then calculated a robust coefficient of variation as 1.4826 * (MAD/ median) * 100 (setting to NA when the median was zero or missing). Figures were created in R using the packages tidyverse v2.0.0 [34], ggplot2 v3.5.1 [38], poisonfrogs v1.0.2 [39] and patchwork v1.3.0 [40].

### Machine learning model establishment, inputs/outputs, and feature selection

Feature selection per outcome variable was initially assessed with an elastic net model with 10-fold cross validation with the glmnet v4.1-8 package [41]. The base morbidity machine learning model used a gradient boosting (gbm) machine algorithm via the gbm v2.2.2 package [42] as previously described [13]. All subsequent development and analyses pertaining to this model were performed in R v4.4.0 using the packages caret v7.0-1 [43], rsample v1.2.1 [44], and fastDummies v.1.7.4 [45], with a split dataset of 70% training and 30% testing using 20x repeated cross fold validation. Balanced accuracy was used to select the best training models while Matthew’s Correlation Coefficient (MCC) was used to assess model performance. The base morbidity model predicts reaching a high weight loss (≥14.5%) of an individual ferret between days 1–14 p.i. as a binary yes/no as previously described [13]. Additional weight metrics (RMSSD, MASD, ACF1) were made into similar binary categories using one third vs two thirds of equally binned data as the split. No models in this study utilized hyperparameter tuning, so that direct relative comparisons between models could be assessed. We further explored weight, temperature, and titer associations by first imputing the means of nasal wash titers for missing days for which each flanking day had values. We then performed linear mixed effects models controlling for day, virus and individual ferret using the lme4 v1.1-35.5 [46], lmerTest v3.1-3 [47], and modelsummary v2.2.0 [48] packages.

## Supporting information

S1 Table Pearson correlations between weight loss and other clinical and virological metrics. (XLSX)

S2 Table Pearson correlations between weight loss (daily AUC) and other virological metrics. (XLSX)

S3 Table Pearson correlations between temperature rise (daily AUC) and other virological metrics. (XLSX)

S4 Table Pearson correlations between weight loss and day of weight loss onset. (XLSX)

S5 Table Pearson correlations between weight loss and persistence of weight loss. (XLSX)

S6 Table Wilcox t tests between lethal outcome and number of days ferrets exhibited weight loss below a defined threshold between days 1–5 or 1–7 p.i. (XLSX)

S7 Table Wilcox t tests between lethal outcome and parameters capturing slope and volatility presented in Figs 3 and 4. (XLSX)

S8 Table Variable importance of different machine learning models using conventional or novel metrics as outcome variable (indicated in bold). (XLSX)

S9 Table Matthew’s Correlation Coefficient for different machine learning models evaluated in this study. (XLSX)
